# Essay on Belladonna

**Published:** 1877-12

**Authors:** J. S. Todd

**Affiliations:** Atlanta, Ga.


ATLANTA
MEDICAL AND SURGICAL JOURNAL

VOL. XV.] DECEMBER—1877. [No. 9

### Original Communications.

### AN ESSAY ON BELLADONNA.

BY J. S. TODD, M.D., ATLANTA, GA.

*Read before the Atlanta Academy of Medicine November 20, 1877.*

The tendency of the world to run after new ideas and dogmas, however absurd, finds no more complete exemplification than the avidity with which medical men seize hold of new remedial agents. Is it that our stock of curatives is so small, or are those we have so little worthy of confidence? Which? The voluminous pages of our Dispensatory answer negatively the first question—the pecuniary success of fluid extract manufacturers of latest discoveries, affirmatively the latter. I very naturally ask what is the cause of our want of faith? It is a fact beyond dispute, that therapeutics has not kept pace with the progress of surgery, pathology and chemistry. Indeed, while pathology has shed a flood of light upon our knowledge of disease, to say the least, it has not accomplished what was expected of it; on the contrary, I am sure that the ardor of the therapeutist has been dulled by the teachings of pathology, which, alas, so often shows that the malady, by its very nature, is incurable. Too much knowledge renders him hopeless of curing with known medicines, and he adds new remedy upon remedy, until their name is legion, in his eager career after potential agents. The search after a universal remedy has been abandoned, but it does seem that the vulgar idea that for every disease there grows some plant that is its antidote, has taken deep hold upon investigators. Have we so few drugs that will serve us when we are in sore distress? Have the potential powers of agents once equal to the emergency been with their advocates consigned to the grave?

This is an age of progress, and I am not one of those who believe that the first Adam was superior in all things to the average man of the nineteenth century; but, on the contrary, am sure that in all the attributes that go to make us more perfect—more like God—we are immeasurably his superior. We know more of the *science* of medicine than did Cullen, Letsom, Abernathy, Physic, or Rush; but I am not certain that we caught their art of administering remedies. The ship of progress, though it may speed on its way ever so rapidly and evenly, has, like every other vessel, in its hold ballast—materialism, if you will—and there is among its freight some article which would with least regret be sacrificed to the angry sea soonest when the storm arises. Of medical goods I opine and hope a large part of the vis medicatrix naturæ—another name, too often, for skepticism about the power of medicine—and stimulation will be first cast overboard. It is not my intention to discourage the investigation of the truth in any of its fields. I say God speed to them all, pathologist among the rest; but if I could put the investigators—three-fourths of them—to studying more closely the action of known remedies, then would my object have been accomplished. I want to increase my understanding of the action of opium, mercury, cinchona, aconite, nux vomica, belladonna, turpentine, cantharides, arsenic, colchicum, and others of this old respectable class of drugs that have stood the test of time. I want Drs. Johnson, Miller, Alexander, or J. G. Westmoreland, to tell us in essays of their first love—that we boys have weaned them off from by *stimulation*—their trusty weapon in the days gone by: *the lancet.* I am sure we would listen; I am sure suffering humanity would not be the loser.

The subject I have chosen is Atropia Belladonna. I selected it because it was of the class of drugs about which so much is known, and—pardon me—so much forgotten: because it has so many virtues unknown, though it has been an agent so long used by our fraternity. If one of us was compelled to confine himself to a dozen remedies, I do not believe that belladonna would be one of them; it is, in fact, a drug not appreciated according to its deserts.

In collecting *facts* regarding its virtues, I have been astonished that we regarded it so lightly. This being true of belladonna—one of our lesser agencies, so considered—what would be our emotions if we studied more closely the recorded *truths* concerning opium or mercury. More experiments with fifteen or twenty old remedies, and less with new ones, will do much to dispel the skepticism that imbues us. The vast importance of atropia as an agent in diseases of the eye, is so well expressed by Dr. Earnest Hart, Opthalmic Surgeon to St. Mary's Hospital, England, in the *British Medical Journal*, April, 1872, that I give it almost entire, feeling sure that to many a country practitioner, where specialists are not to be found, it will be useful.

Dr. Hart does not think he shall be using an exaggerated form of expression, or going beyond the strict and well balanced weight of words which is necessary to give due force to the fact to be conveyed, if he says that we could, in the treatment of opthalmic disease, better afford at this day, so far as our knowledge of disease and means of mastering it extends, to dispense with all other drugs, lotions, and applications put together, than with this one topical medicament. Let us consider what atropine does for the inflamed eye. It allays local sensitiveness, and removes local spasm; it gives to the eye and internal muscular apparatus—iris and ciliary muscle—physiological rest, the greatest of all curative means. Nor does it do this only, but, in dilating the pupilary aperture, it drives far from us the bug-bear which long haunted the opthalmic surgeon, and which still pursues those who are not sedulously active in the use of atropine—closure of the contracted pupil by an adherent plug of lymph, and gluing of the posterior surface of the iris to the lens. It would rob the consulting surgeon of a great many profitable but trying operations, if the atropine eye-drops were ready in every surgery, not only on all emergencies, but for the exigencies of daily practice. It is as safe a rule in opthalmic practice to use an atropine drop, when in doubt, as in whist to play a trump. Dr. Hart can hardly think of more than one absolute contra-indication, and that is the existing oval dilatation of the iris in glaucoma. There are, of course, also mechanical contra-indications, as in peripheral wounds of the cornea with hernia of the iris, when to dilate the iris is to increase the peripheral protrusion; but, even here, the moment the corneal gap is healed, atropia becomes of the first necessity. But in all cases of iritis, in contusions and injuries of the eye, in corneitis, purulent ophthalmia, scrofulous ophthalmia, and deep-seated, mixed inflammations of the eye, the local application of a solution of atrophia is the most precious of therapeutic means. * * * * With a little cotton wool, alum, and glycerine, hot and cold water and atropine, and a pocket case of instruments, we can treat, with a previously unattainable success, nearly the whole range of opthalmic diseases. * * * It is possible, but not easy, to abuse atropine. His formula for its use, is—

| | |
|---|---|
| ℞.—Atropia sulphate, . . . . . | grs. ij. |
| Glycerine, . . . . . . . | gts. v. |
| Aqua distilled, . . . . . . | ℥ j. |

When the sulphate cannot be procured, a drop or two of acetic acid will be necessary to cause the alkaloid to make a perfect solution in water.

As a remedy for constipation, extract of belladonna, given before breakfast, in one-fourth grain doses, possesses advantages not sufficiently appreciated. It does not gripe, nor does it interfere with digestion; it is easy of administration, and causes semi-solid evacuations, as healthy in appearance as those produced from any other laxative. Dr. Morris, in the *London Lancet*, relates four cases of intestinal obstruction which resisted every remedy which could be devised. Thinking the obstruction might be caused by violent contraction of the circular muscular fibers of the bowels, two grains of extract of belladonna were passed up the bowels, and in about two hours a copious discharge of hard scybala followed, with a cessation of all the distressing symptoms consequent on the obstruction.

Prfessor Bartholow, of Ohio Medical College, who has recently written the best treatise extant on therapeutics, and is, withal, one of the most profound thinkers of the age, in a prize essay written in 1872, and read before the American Medical Association, on atropia, called attention to the usefulness of this agent in allaying the night-sweats of phthisis. This discovery has been appropriated very coolly by Dr. Ringer, of England. Dr. Fothergill, in the *Practitioner*, 1876, giving his experience with atropine in the treatment of night-sweats, says that "the public, as well as the profession, are under a deep debt of gratitude to Dr. Ringer, for this discovory." That atropine, or belladonna, does possess the power of curing night-sweats, or hydrosis of any kind, is, beyond a doubt, the fact, having been fully demonstrated by numerous competent witnesses. Belladonna seems to be a specific anhidrotic, (an agent that prevents profuse perspiration) acting on the sudoriferous glands as it does on the submaxillary gland. I know, by practical experience in several cases, that there is no agent that will afford so speedy and marked relief in ptyalism as small doses of this drug—say from one-sixth to one-fourth grain of the extract of belladonna *ter die.* I would suggest that a trial be given the drug in follicular pharyngitis, when the secretion is excessive, and in chronic laryngial and pharyngial inflammations generally, where the secretion of mucus was too great.

As a remedy for incontinence of urine, or where there is irritability about the neck of the bladder, I know of nothing which has given such gratifying results in my hands. Two years ago a member of the Academy casually mentioned his success with belladonna in the malady above mentioned. It caused general surprise, the novelty of the idea. I was among those to whom this virtue of the drug was new, and yet in the dispensatories out of date, it is advised that it be given where "there is incontinence of urine, spasmodic stricture of the urethra, or neck of the bladder." This is an apt illustration of our inexcusable ignorance of the virtues and potencies of drugs which have served us from time immemorial. Belladonna has long been recognized one of our best agents to arrest the lacteal secretion, either given internally, or rubbed on the areola of the breast, or applied to the gland as a plaster. This local action of belladonna directed the attention of Dr. Sidney Ringer, F.R.C.P., to its effects as a local application, either in liniment or ointment. He first employed it "in a case of unilateral sweating of the right side of the neck and face, breaking out upon the slightest exertion or excitement, or when near a fire, so that the sweat ran down his face and neck in streams, soaking his collar and the band of shirt, his face being neither red nor congested;" the application of the belladonna liniment, in a short time effected a cure. The writer also mentions many cases cured by its local application, in young children, where the copious sweating of their head and face is aften so profuse as to wet their hair, and the pillow on which they are sleeping. Of course, with so poisonous a drug, much precaution is necessary, that we be careful not to apply, if there are abraded surfaces, or in so great a quantity that its absorption by the skin may produce alarming symptoms. Topically to sweaty hands and feet it has wrought many cures according to the same authority.

As a local application to rigid *ores tincarnum*, to swelled testicles, to strangulated hernia and indurated glands, it is one of the orthodox agents. Its power of doing good in obstinate intermittents, where the nervous system has become affected, though often mentioned by writers, is too little regarded. Its prophylactic power over scarlet fever, while not proven, is asserted by many to be very great. For myself, I will say that the probabilities of its doing what is claimed for it as a preventive of this terrible disease, is, in my mind, so fixed that I shall always avail myself of it. The possibility of its causing harm in the dose recommended—one drop *ter die*, of a tincture made from one grain to two grains of the extract to one ounce of alcohol (Dr. Flint)—is not a question for serious consideration. While I do not believe that belladonna is an antidote to opium, candor compells me to admit that the testimony in favor of its antidotal power in cases of opium poisoning, has decided preponderence, so much so that I would probably not be justified, if I withheld it in a case terminating fatally.

Recently arsenic and belladonna are attracting the favorable attention of profound thinkers in the cure of phthisis. So much so that Dr. Fothergill—one of *the* eminent men in our profession—says, "I have no hesitation in saying that the use of this agent (atropia) completely changes the aspect of many cases of pulmonary phthisis. It has been much more cheering since I employed belladonna extensively." Prof. Bartholow, of Ohio, in an exceedingly interesting article in the *American Journal of Medical Sciences*, April, 1877, "On the treatment of certain forms of phthisis pneumonalis by rest and the internal administration of atropine," gives in full the history of two cases, one of which is so instructive that I synopsize it: Mrs. R., æt. 25; married; weight 95 pounds; slight figure; narrow chest; three sisters and both parents had died of phthisis; cough for several years, which was now almost constant; depression extreme; took but little food; hectic; profuse night-sweats; menstrual flow ceased; temperature habitually above 100 degrees; in the evening 102 degrees; respiration 40; expectoration purulent. Physical examination showed consolidation of superior lobe of left lung, softening and extrusion, with cavities; commencing deposit in apex of right lung. She spent the most of her time confined to her room or bed. Treatment—a solution of atropia, grs. j to ℥ij of water, of which she took two minims, morning and evening. The prescription was first made in 1867, and owing to a misunderstanding she took it in increasing quantity for two years. He was several times summoned to see her, during this period, in consequence of decidedly toxic symptom. An improvement which is only characterized by the word wonderful has occurred; the cough lessened; the sweating and fever ceased; the menses reappeared; she became enciente; during that time appeared in vigorous health; was delivered of a healthy child in 1871, and now, ten years after he first saw her, she is in excellent health; weighs 115 pounds, etc. In the other case, he was equally as successful, and eight years has elapsed since treatment was begun. He says he has quite a number of cases, but withholds their publication until a sufficient time elapses after treatment to make them trustworthy.

Alcohol has long been considered one of the best remedies for phthisis. An analogy between the action of alcohol and belladonna is not difficult. According to Headland, (one of the very best authorities on the action of medicine) alcohol and belladonna are classed as narcotics—the first an inebriant narcotic, the last a delirient narcotic. As a direct cardiac stimulant, Dr. Horatio Wood, Jr., of the University of Pennsylvania, says no agents, not even ammonia or alcohol, are so powerful as minute doses of atropine. It is, therefore, one of our best remedies in all cases of poisoning or accident, where death is imminent from failure of the heart's action.

As an adjuvant in the treatment of nervous disorders, especially neuralgia, belladonna has long been favorably known. As a substitute for opium it will almost always disappoint; but the uses to which belladonna, in conjunction with opium, can be put, that will materially enhance both, are so many, that an article on either, would be incomplete if they were omitted.

Remember that the belladonna causes delirium, hallucination, etc., while the other, opium, stupor, quietude, sleep, and entire relief from pain; combine the two, and the latter, cessation from pain—the pleasantest and most thankful office of the physician—can be procured, and still your grateful patient can, with intellect wide awake, discharge his ordinary duties.

Opium depresses the action of the heart; atropia stimulates it; between the two, generally, the cardiac impulse will not be affected. Belladonna is more lasting in its action than opium, and this virtue of the former materially lessens, sometimes completely obviates, the depression, nausea, and dullness that follows an hypnotic dose of the latter. The pain-relieving power of opium is enhanced by atropia. Both produce dryness of the mucous membrane of the mouth and fauces. Morphia suspends, atropia increases peristatic movements. Morphia increases the action of the sudoriferous glands, atropia lessens it; in combination the action of the two on the kidneys is to increase the flow of urine.

It seems to be the general verdict of late experimenters that two doses a day of atropine is all that is requisite, as it is very lasting in its action. As its employment in the sweating of phthisis—though established as one of our best agents—is somewhat new, it will probably be well that I give the general dose adopted in the London Hospitals. Dr. Ringer gives from $\frac{1}{200}$ to $\frac{1}{100}$ of a grain of atropine, hypodermically, and from the $\frac{1}{80}$ to $\frac{1}{50}$ per orem. Dr. Fothergill, who states that he had, at the time he wrote the article from which I quote, one hundred patients in the various hospitals in London, on atropine; that, while he has no experience in the drug given hypodermically, he commences, per orem, on the $\frac{1}{75}$ and gradually increases to the $\frac{1}{25}$ if necessary, the latter dose rarely failing. After the sweats are broken, the minimum dose will prevent their return, and generally the patients will not complain of the toxic action. If the atropine or belladonna purge, the union of $\frac{1}{2}$ to 1 grain of aloes to each dose will obviate this effect. The cases most favorably influenced by it, says Dr. Fothergill, are those who have a fast pulse, above 100, a temperature at and above 100 degrees Fahrenheit, cough, profuse night-sweats, and rapid wasting. In conclusion, I think I hazzard nothing in asserting that if half the testimony I have adduced in favor of atropine, could be gotten for fluid extract of grindelia robusta, yerba santa, glycerite of kephaline, and other new drugs, in the treatment of phthisis, there would be no difficulty in having their virtues thoroughly tried. Indeed, their favorable consideration and general testing would not require such names as Bartholow, Ringer, or Fothergill.

NOTE.—After my Essay had been read and discussed by the members of the academy, it was requested that I go more fully into the theory of the action of belladonna. The paper was only intended to excite an interest in a drug that was looming up so prominently, and to do this, I thought *facts* were worth more than theory; and, beside, the usual length of such papers forbade anything like an exhaustive treatise. A reference to almost any standard work on therapeutics, will give in full its action better than I, with my inexact ideas, would presume to essay. "Blessed is he who knows the reason of things, for such knowledge is not superficial," is an aphorism, which, if rigidly applied to our understanding of the action of medicines, the why and wherefore, would in the large majority of instances, inevitably lead to the conclusion that our information was superficial. Nevertheless, we *know* that opium relieves pain, sulphur kills the acarus, quinine cures ague—how, we will probably always be left to conjecture.

To describe the action of hæmatirics, catalitics, astringents or alteratives, is easy, in comparison with any attempt to give the modus operandi of neurotics. "When we consider that little or nothing is known, or can be known, about the ultimate cause of sensation or motion or nervous excitement, there is no need for wonder that we find ourselves at a loss to explain the operation of medicines that influence these conditions." For these very cogent reasons, I confined myself to *what* belladona did, deeming it almost impossible to tell *how*. But as the request was preferred, I will say something about the physiological action, &c.

Many theories have been enunciated to account for its action in dilating the pupil. The one which seems to my mind better suited to explain, not only this effect of the drug, but also its cathartic action and undoubted power of relieving pain, when locally applied, is this: It paralyzes the nerves—ciliary branches of the sympathetic system—of pupilary accommodation. Dr. Harvey contends that it causes dilitation by stimulating the radiating fibres of the muscular apparatus of the iris.

I cannot subscribe to this theory, for several reasons—among them, light is a stimulus to the pupil, causing it to contract, while darkness being the contrary, causes dilatation. And again, I believe its action is on the nervous system, not the muscular or arterial, except *a priori*. The dryness, so characteristic in the throat and fauces from belladonna, is, doubtless participated in by the stomach and bowels, but this condition is in them mere transitory, and increased secretion quickly followed by catharcis, is the result. Given to persons of light complexion, especially women, a difiuse redness of the skin, bearing very near resemblance to scarlitana, is occasioned. This shows that it is a stimulant to cutaneous circulation; hence, the rationale of its curing night-sweats. But here it might be very reasonably urged by those who maintain that it dilates the pupil by stimulating the muscular fibres *per se*, that the redness of the skin was confirmatory. The impossibility of getting a theory to fit all actions of any neurotic, is apparent. Ergot, we know, causes uterine contractions—increased *muscular* action of that particular organ—yet the heart, a large, strong muscle, is so little interfered with that it escapes notice in the large majority of cases where ergot has been given. The leaves of the deadly night-shade, steeped in water and applied to cancerous ulcerations, was at one time esteemed among the best agents known to relieve the pains of carcinomatous ulceration, and the practice has been discontinued only because we could not regulate the amount that would be absorbed, hence toxic symptoms were not infrequent. But how does it relieve pain? Here another lion is in the path. What is pain, and where is its hiding place? What causes it? In this case—the cancer—I think the pain is caused by muscular contractions—spasmodic action of those muscles or fibres supplied by sympathetic nerve filaments. The atropia here, as in the iris, *through the nerves*, causes paralysis and cessation of motion in the muscles, and pain, as a consequence, ceases, the cause being removed.

Reference was made in the body of this paper to small doses of aloes preventing the cathartic action of the extract of belladonna. This may appear paradoxical, like two wrongs making a right; for two cathartics given in combination counteracting each other, is, I must confess, like two negatives being equalled to a positive. But experience, that fell, ruthless puller down of the finest spun theories, says it is a *fact*, and all facts have reasons for their action. I think I can, if you will bear with me, reconcile these apparent contradictions. To do this, we must know that aloes expends its *principal* force on the large intestines, the peristatic contractions of which it increases by irritating the mucous membrane, the nerves of which supply their muscular coat. Now, these very nerves are to a certain degree paralyzed by belladonna; increased patulence of the intestinal tube resulting from the paralysis, permits of more rapid movement of the ingesta: give aloes in small doses, and the irritation it occasions prevents this peculiar action of belladonna, and vice versa. The result is, no disturbance—catharsis is not occasioned.

Narcotics, for convenience, may be divided into three classes—somnolant, inebriant and delirient. Opium, a representative of the first, causes sleep, but leaves the mind free, for we dream while under its influence. Alcohol, which is typical of the second, in full or toxic dose, causes drunkenness, which is a cessation of volition, motion and intellection. Belladonna, a head representative of the last division, while paralyzing slightly the senses and motion, leads all astray, so to speak. It causes delusions, hallucinations and busy delirium; rarely somnolence. Hence, it should be withheld in delirum, congestion of the brain or cord, &c. It increases the number of respirations, and for this reason, mainly, has been urged as an antidote to opium. Voluntary muscular motion is not affected by atropia.

These desultory remarks on the physiological action of atropia are as incomplete, I feel sure, as were those on its known effects; but if they excite sufficient interest to cause a study of the many excellent monograms that have been written on atropia belladonna, I will be repaid for my labor.

